# Deletion of Batf3-dependent antigen-presenting cells does not affect atherosclerotic lesion formation in mice

**DOI:** 10.1371/journal.pone.0181947

**Published:** 2017-08-03

**Authors:** Jesus Gil-Pulido, Clement Cochain, Malte A. Lippert, Nicole Schneider, Elke Butt, Núria Amézaga, Alma Zernecke

**Affiliations:** Institute of Experimental Biomedicine, University Hospital Würzburg, Würzburg, Germany; Universiteit van Amsterdam, NETHERLANDS

## Abstract

Atherosclerosis is the main underlying cause for cardiovascular events such as myocardial infarction and stroke and its development might be influenced by immune cells. Dendritic cells (DCs) bridge innate and adaptive immune responses by presenting antigens to T cells and releasing a variety of cytokines. Several subsets of DCs can be discriminated that engage specific transcriptional pathways for their development. Basic leucine zipper transcription factor ATF-like 3 (Batf3) is required for the development of classical CD8α^+^ and CD103^+^ DCs. By crossing mice deficient in *Batf3* with atherosclerosis-prone low density lipoprotein receptor (*Ldlr*^*-/-*^*)*-deficient mice we here aimed to further address the contribution of Batf3-dependent CD8α^+^ and CD103^+^ antigen-presenting cells to atherosclerosis. We demonstrate that deficiency in Batf3 entailed mild effects on the immune response in the spleen but did not alter atherosclerotic lesion formation in the aorta or aortic root, nor affected plaque phenotype in low density lipoprotein receptor-deficient mice fed a high fat diet. We thus provide evidence that Batf3-dependent antigen-presenting cells do not have a prominent role in atherosclerosis.

## Introduction

Cardiovascular diseases constitute a major public health concern and represent the leading cause of death in Western societies. Regarded as a chronic inflammatory disease of the vessel wall, atherosclerosis is the main underlying process responsible for events such as myocardial infarction and ischemic stroke. It is initiated by a dysfunction of the vascular endothelium, the cellular lining of the vessel wall, and changes in permeability, promoting uptake and retention of circulating lipids, such as cholesterol-containing low-density lipoproteins (LDLs). In consequence an upregulation of adhesion molecules leads to the recruitment of circulating immune cells [1]. LDL particles can be oxidized (oxidized-LDL, oxLDL) and engulfed by aortic macrophages, which become engorged with lipids to histologically appear as foam cells [2]. Macrophages constitute the major cell population within plaques. However, also other immune cells, such as T cells [3], B cells [4] and dendritic cells (DCs) [5] are present within atherosclerotic lesions and have been shown to contribute to disease development.

DCs serve as a bridge between innate and adaptive immune responses by engulfing, processing and presenting antigens to T cells. In the context of atherosclerosis they can impact disease progression by affecting lipid metabolism, either by locally taking up oxLDL to form vascular foam cells [6] or by promoting lipid clearance from the circulation [7]. Furthermore, DCs are able to polarize T cell responses [7]. In previous studies, however, mostly promiscuous surface markers were used to identify DCs, such as CD11c, which can also be expressed by activated monocytes/macrophages [8] so that a clear attribution of the effects observed to *true* DCs is difficult and the less specific term of antigen-presenting cells should have be applied.

Different subsets of *bona fide* DCs, i.e. plasmacytoid DCs (pDCs) and classical DCs (cDCs) that can further be segregated into CD8α^+^ cDCs in lymphoid and CD103^+^ cDCs in non-lymphoid tissue, and CD11b^+^ cDCs can be discriminated, that engage specific transcriptional pathways for their development. Basic leucine zipper transcription factor ATF-like 3 (Batf3) belongs to the activator protein 1 (AP-1) family of transcription factors. It is known to be a master transcription factor for the development of classical CD8α^+^ and CD103^+^ DCs, and is expressed at low levels or absent in other immune cells [9, 10]. Both CD8α^+^ and CD103^+^ DC subsets have been ascribed an important role in cross-presentation, a process by which DCs present external antigens by MHC-I molecules to induce CD8^+^ T cell responses [11].

The role of Batf3-dependent APCs in the development of atherosclerosis is not clear. In a study in which lethally-irradiated low density lipoprotein receptor-deficient (*Ldlr*^-/-^) mice were reconstituted with bone marrow (BM) from *Batf3*^-/-^ mice and as a consequence lacked CD8α^+^ DCs, no differences in plaque development were observed [12]. More recently, a study using Apolipoprotein E-deficient (*ApoE*^*-/-*^) *Batf3*^*-/-*^ mice has proposed that Batf3-dependent DCs promote atherosclerosis through induction of Th1 responses in the aorta [13]. In another study, using FMS-like tyrosine kinase 3 ligand (Flt3)-deficient *Ldlr*^-/-^ mice, a specific loss of CD103^+^ DCs was noted in the aorta. In these *Flt3*^-/-^*Ldlr*^-/-^ mice, the loss of CD103^+^ DCs was associated with a reduction in regulatory T cells (Treg) in the aorta, so that CD103^+^ DCs were ascribed atheroprotective roles [14]. Finally, a recent report has investigated the function of a subset of CD8α^+^/CD103^+^ DCs expressing C-type lectin receptor (Clec9a) or DNGR1. Absence of DNGR1 was shown to reduce atherosclerosis development in a context of moderate hypercholesterolemia, associated with an increase in the expression of interleukin-10 (IL-10) [15].

Bone marrow transplantation models are frequently used to study the contribution of hematopoietic cells to atherosclerosis. However, irradiation that is required to deplete the hematopoietic compartment of recipient mice has been reported to have an intrinsic effect on the progression of atherosclerosis [16], and effects on atherosclerosis can differ between chimeric *Ldlr*^-/-^ or apolipoprotein E knock out (*Apoe*^-/-^) mice when compared with findings obtained by using full knock outs. For instance, *Ldlr*^-/-^ mice that lack B7-1/B7-2-T cell-costimulatory signals showed less atherosclerosis due to decreased T cell activation [17], whereas irradiated *Ldlr*^-/-^ mice reconstituted with bone marrow cells lacking B7-1/B7-2 had increased plaque area through decreased Treg responses [18].

It is not known if irradiation affects resident aortic cells, such as CD103^+^ DCs. To decipher the role of Batf3-dependent APCs in atherosclerosis while avoiding biases associated with the use of bone marrow chimeric models we here have used *Batf3*^-/-^ mice that lack CD8α^+^ and CD103^+^ APCs and crossed these with *Ldlr*^-/-^ mice to obtain *Ldlr*^-/-^*Batf3*^-/-^ knock out animals. We show that full Batf3 deletion did not affect the development of atherosclerosis or alter plaque compositions and was accompanied by only minor changes in the immune response. Thus, we conclude that Batf3-dependent APCs have no major function in atherosclerosis.

## Materials and methods

### Mice and diet

Basic leucine zipper transcription factor ATF-like 3-deficient (*Batf3*^-/-^) mice were crossed with *Ldlr*^-/-^ mice (both C57BL/6J background, Jackson Laboratory) to generate *Ldlr*^-/-^*Batf3*^-/-^ mice. Mice were bred and housed under specific pathogen-free conditions maintained on a standard light-dark cycle, and *ad libitum* access to food and water. For atherosclerosis induction, male or female mice aged 6 or 8 weeks, were grouped in cages (maximum 3 mice per cage) and placed on a high fat diet (HFD) (15% milk fat, 1.25% cholesterol, Altromin, Germany) for 8 and 12 weeks, respectively. Animals on diet were inspected once daily. At the end of the study, mice were anesthetized using isoflurane and euthanized (ARRIVE Guidelines Checklist in S1 File). All animal studies and numbers of animals used conform to the Directive 2010/63/EU of the European Parliament and have been approved by the appropriate local authorities (Regierung von Unterfranken, Würzburg, Germany, Akt.-Z55.2-2532-2-82).

### Flow cytometry

For FACS analyses, tissues were disrupted and passed through a 70 μm filter (BD Biosciences, Germany) to obtain single-cell suspensions. For aortic sinus analysis, fat was carefully removed from the aorta and ascending and descending aorta was separated from the aortic root. They were minced and incubated 1 hour at 37°C with 450 U/ml Collagenase I (C1030), 125U/ml Collagenase XI (C7657) and 60U/ml of hyaluronidase (H3506) (all from Sigma Aldrich). Whole blood was combined with a red blood cell lysis buffer (155 mM NH4Cl, 10 mM KHCO3, 0.1 mM EDTA) to allow the isolation of leukocytes. For dendritic cell studies cell suspension were first incubated 20 minutes with anti-Fc receptor (CD16/32) at 4°C to avoid unspecific bindings and processed as described next. Cells were stained for 30 minutes on ice using combinations of specific antibodies from BD biosciences (CD45, clone 30-F11; CD3, clone 500A2; CD8a, clone 53–6.7; Ly6G, clone 1A8; CD11b, clone M1/70; CD4, clone RM4-5), eBioscience (TCRβ, clone H57-597; CD44, clone IM7; CD4, clone GK1.5; Foxp3, clone BM8; CD25, clone PC61.5; IL-17a, clone eBio17B7; CD86, clone PO3.1; CD11b, clone M1/70; CD11c, clone N418; SiglecH, clone eBio440c; MHCII, clone M5/114.15.2; CD115, clone AFS98; Ly6C, clone HK1.4; TCRγδ, clone eBioGL3; IFNγ, clone XMG1.2; CD103, clone 2E7) and Biolegend (CD62L, clone MEL-14; CD16/32, clone 93). All antibodies were used at 1:300 dilution except for anti-CD103, which was used at 1:100. Intracellular staining was performed using the Cytofix/Cytoperm solution (BD Biosciences) on cells treated with 50 ng/ml PMA, 750 ng/ml ionomycin and 2.5 μg/ml brefeldin A for 4 hours (all from sigma Aldrich). Intracellular labeling of Foxp3 was performed using the Foxp3 Staining Buffer Set (eBioscience) according to the manufacturer’s instructions. Probes were analyzed using a FACSCanto II (Becton Dickson, USA) and FlowJo 10.0 software (Treestar Inc., USA).

### Immunohistochemistry and atherosclerotic lesion quantification

Arteries were perfusion-fixed in situ with phosphate buffered saline (PBS) followed by 4% paraformaldehyde in PBS (PFA; Sigma Aldrich, USA). The heart and whole aorta were removed and carefully cleaned of extraneous fat before being post-fixed in 4% PFA. The heart was embedded into paraffin and cut into 5-mm transverse sections. Aortic root sections were assessed for atherosclerotic plaque size after staining with Gabe’s Aldehyde Fuchsin. Adjacent sections were used to assess plaque cellular content by immunofluorescence staining of macrophages by mAb staining for Mac2 (rat anti-mouse, Cedarlane, Canada). Briefly, slides were blocked with 1% bovine serum albumin (Sigma Aldrich), incubated with primary antibody overnight at 4°C, and secondary detection using the relevant Alexafluor 488-conjugated antibody (Molecular Probes, Life Technologies, Germany). Sections were coverslipped using DAPI-containing Vectashield mounting medium (Vector laboratories, Burlingame, USA). The extent of atherosclerosis throughout the aorta was assessed by staining for lipid depositions with Oil-red-O. Briefly, the aorta was opened longitudinally, stripped of adventitia and the percentage of lipid deposition was calculated by dividing the stained area by the total aortic surface. All images were recorded with a Leica DM 4000B fluorescence microscope and JVC KY-F75U camera. Plaque size, collagen content and cell content were quantified by computerized image analysis (Diskus Software, Hilgers, Germany) and by investigators blinded to the group distributions.

### Serum cholesterol and triglyceride measurement

Serum was analyzed for total cholesterol (Amplex Red Cholesterol Assay Kit, Invitrogen, Life Technologies) and triglycerides (EnzChrom Trigliceryrides Assay Kit, BioAssay Systems, USA) according to the manufacturer´s instructions.

### Serum lipoprotein profile

Serum cholesterol lipoprotein profiles were determined by size exclusion chromatography. In brief, 5 μl of serum was fractioned using a Superose 6 3.2/300 gel filtration column from GE Healthcare (Uppsala, Sweden) and PBS, pH 7.4 as elution buffer, delivered by a first pump (Waters 1525 binary pump, Eschborn, Germany) at a flow rate of 50μl/min. The separated lipoproteins were mixed in a T-tube with 50μl/min cholesterol reagent (Roche, Mannheim, Germany) delivered by a second pump (Waters 1515 pump). Thereafter, the mixture went through a 500 μl reaction coil PEEP tubing (internal diameter 0.75mm) at 37°C in a post column reaction oven (Waters Temperature Control Module II). Finally, absorption was measured with an UV-VIS detector at 500nm (Waters 2489 UV7Visible Detector). Total run time for each sample was 60 min. Chromatograms were integrated by Waters Empower 3 software. Very low density lipoprotein (VLDL), LDL and high density lipoprotein (HDL) concentrations were calculated as products of the area percent of total cholesterol.

### Generation of mouse bone marrow-derived macrophages (BMDMs)

BMDMs were obtained from *Ldlr*^*-/-*^ and *Ldlr*^*-/-*^*Batf3*^*-/-*^ as described previously [19]. Briefly, femurs were removed from donor mice, the bone marrow was flushed and 10^6^ cells were cultured in 24-well plates in RPMI-1640 (with 2mM L-Glutamine, Gibco, Grand Island, NY, USA) containing 10% heat-inactivated fetal calf serum, 100U/ml Penicillin-Streptomycin and β-Mercaptoethanol (50μM, Gibco) supplemented with 15% L-929 cell-conditioned medium [20]. Medium was renewed at days 3 and 5. BMDMs were used at day 7.

### Foam cell formation

To induce foam cell formation, BMDMs were first starved with RPMI-1640 containing 2% BSA for 4 hours and then washed once with warmed PBS. Using the same medium cells were stimulated with 50μg/mL of oxLDL (Hycultec, Beutelsbach, Germany) and cultured overnight at 37°C. Cells were then detached using StemPro^TM^ Accutase^TM^ Cell Dissociation Reagent (Thermo Fisher Scientific, Massachussetts, USA), fixed with BD CytofixTM (Becton Dickson, USA) for 5 minutes at room temperature followed by staining with Nile Red (Sigma Aldrich, USA) diluted 1:2000 in PBS for 10 minutes. After two washes with PBS cells were measured by flow cytometry.

### RNA isolation and quantitative PCR analysis

Total RNA was isolated from BMDMs using the NucleoSpin^®^ RNA kit (Macherey-Nagel, Düren, Germany) according to the manufacturer´s recommendation. cDNA (100ng) was reverse transcribed from total RNA using the first strand cDNA synthesis kit (Fermentas, St. Leon-Rot, Germany). Quantitative real-time PCR analysis was performed from cDNA (10ng) using SYBR green mix (Fermentas) and mouse gene specific primer sets (Hprt Forward: 5´-TCCTCCTCAGACCGCTTTT-3´ and Reverse 5´-CCTGGTTCATCATCGCTAATC; Batf3 Forward: 5´-TCCACGAGGAGCACGAGA3´-, Reverse 5´-CCACATGTACCCCTGGACAC-3´; Tbet Forward: 5´-GCCAGGGAACCGCTTATATG-3´, Reverse 5´-GACGATCATCTGGGTCACATTGT-3´; RORγt: Forward 5´-TGAGGCCATTCAGTATGTGG-3´, Reverse: 5´-CTTCCATTGCTCCTGCTTTC-3´) and a QuantiStudio 6 Flex Thermal cycler (Applied Biosystems, Foster City, CA, USA).

### Statistical analysis

Data represent mean ± SEM. Gaussian distribution of values was checked using D’Agostino and Pearson Normality test. When normality test was passed for values from both experimental groups, an unpaired t test was performed. When normality test failed, a non-parametric Mann-Whitney U test was performed. Differences where P<0.05 were considered to be statistically significant.

## Results

### *Batf3* deficiency specifically depletes CD8α^+^ and CD103^+^ APCs in lymphoid organs and the aorta *of Ldlr*^-/-^ mice

Batf3 has been shown to be a key transcription factor for the development of CD8α^+^ and CD103^+^ classical DCs without affecting other immune cell populations, including macrophages [10, 21, 22]. In line with previous reports [10], spleens from *Ldlr*^-/-^*Batf3*^-/-^ mice showed a dramatic decrease in CD8α^+^ APCs frequencies, as analyzed by flow cytometry (Panels A and B in S1 Fig). No differences in pDCs were noted (0.37% ± 0.08% *versus* 0.32% ± 0.03% CD11c^low/+^ Siglec H^+^ cells among CD45^+^ cellls in *Ldlr*^-/-^ *versus Ldlr*^-/-^ *Batf3*^-/-^ mice, respectively). CD103^+^ DCs can be found within the aortic sinus of chow-fed animals where they represent up to 20% of total DCs [14, 23]. As expected, CD103^+^ APC were completely absent in cell suspensions prepared from the aortic sinus of *Ldlr*^-/-^*Batf3*^-/-^ mice compared to control *Ldlr*^-/-^ animals, whereas a relative increase in CD11b^+^ APCs were observed (Panel C in S1 Fig).

### CD8α^+^ and CD103^+^ APCs do not impact the development of atherosclerosis

**Fig 1:**
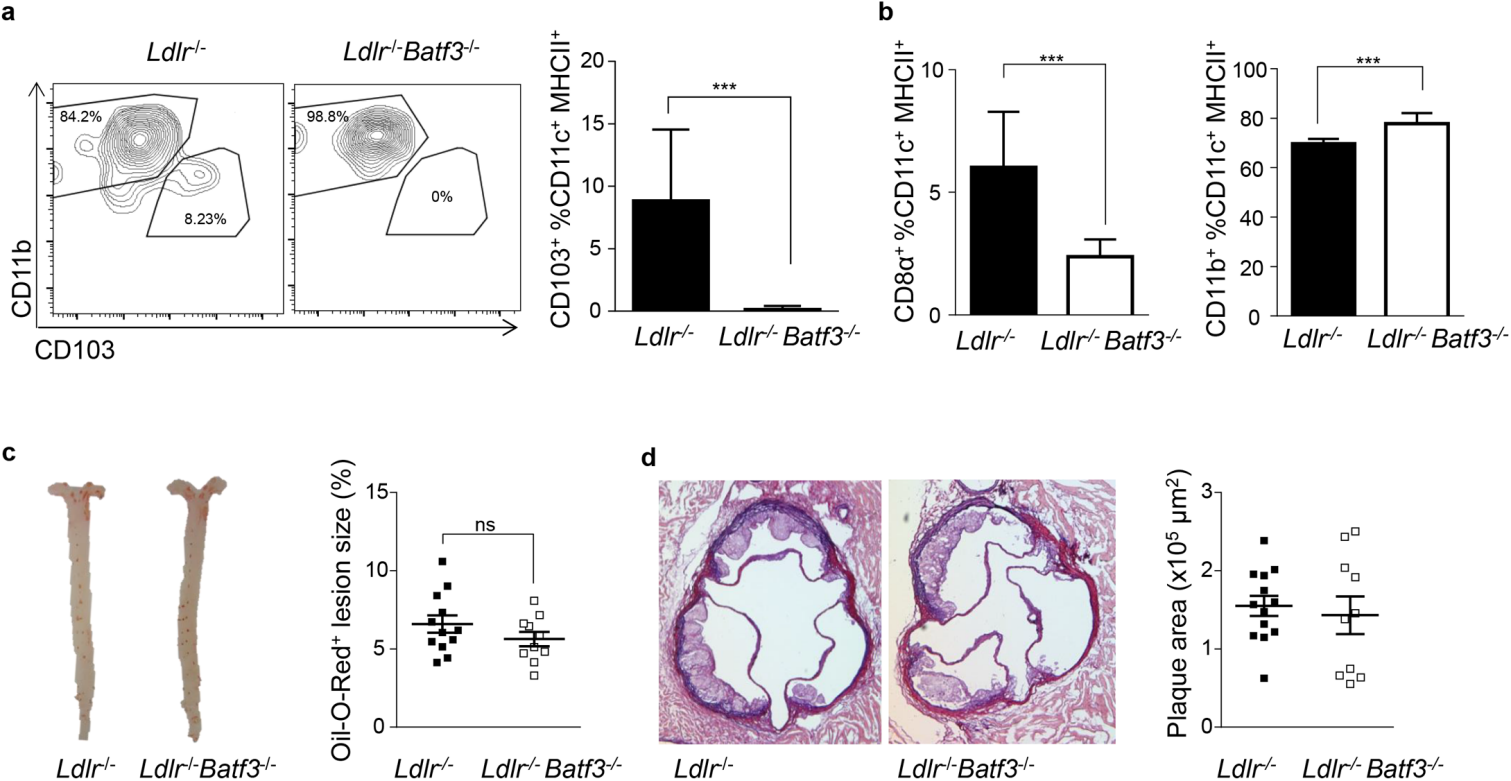
Deficiency in *Batf3* does not alter atherosclerotic lesion development. (a,b) Single cell suspensions from aortae and splenocytes were obtained from *Ldlr*^-/-^ and *Ldlr*^*-/-*^*Batf3*^*-/-*^ mice fed a HFD for 8 weeks and analyzed by flow cytometry. CD11c^+^ MHCII^+^ DCs were gated and further discriminated by expression of CD11b and CD103 in the aorta (a) or CD8α in the spleen (b). (c-d) Quantification of plaque area in Oil-Red-O stained aortae and in Aldehyde Fuchsin-stained aortic roots in atherosclerotic *Ldlr*^-/-^ (n = 12) and *Ldlr*^-/-^*Batf3*^-/-^ mice (n = 10) fed a HFD for 8 weeks; representative images of the aorta and aortic root sections are shown. Data ara presented as mean ± SEM; ns, non significant.

To determinate whether lack of CD8α^+^ and CD103^+^ APCs in *Ldlr*^-/-^*Batf3*^-/-^ mice affects the development of atherosclerosis, male *Ldlr*^-/-^ and *Ldlr*^-/-^*Batf3*^-/-^ mice were fed a cholesterol-rich diet. After 8 weeks of diet, we could confirm the selective decrease in CD103^+^ and CD8α^+^ APC populations in *Ldlr*^*-/-*^*Batf3*^*-/-*^ mice in the aorta and spleen respectively (Fig 1A and 1B). We then analyzed atherosclerotic lesion development in the aorta and aortic sinus. Lesion size was similar in *Ldlr*^-/-^*Batf3*^*-/-*^ animals as compared to controls (Fig 1C and 1D). We did not observe any differences in body weight, serum cholesterol or triglycerides levels (Table 1), and no differences in VLDL, LDL or HDL lipoprotein profiles were noted between groups (Panel A in S2 Fig), which suggests that loss of Batf3-dependent APCs does not affect lipid levels in atherosclerotic *Ldlr*^-/-^ mice.

**Table 1:** Lipid levels of *Ldlr*^*-/-*^ and *Ldlr*^*-/-*^*Batf3*^*-/-*^ mice fed a high fat diet for 8 weeks.

	*Ldlr*^-/-^	*Ldlr*^-/-^ *Batf3*^-/-^	p-value
**Body weight (g)**	27.4 ± 1.6	28.9 ± 1.3	0.24
**Serum cholesterol (μg/ml)**	23378 ± 4656	21533 ± 6962	0.46
**Serum triglycerides (mmol/L)**	4.7 ± 1.2	3.7 ± 1.0	0.06

**Fig 2:**
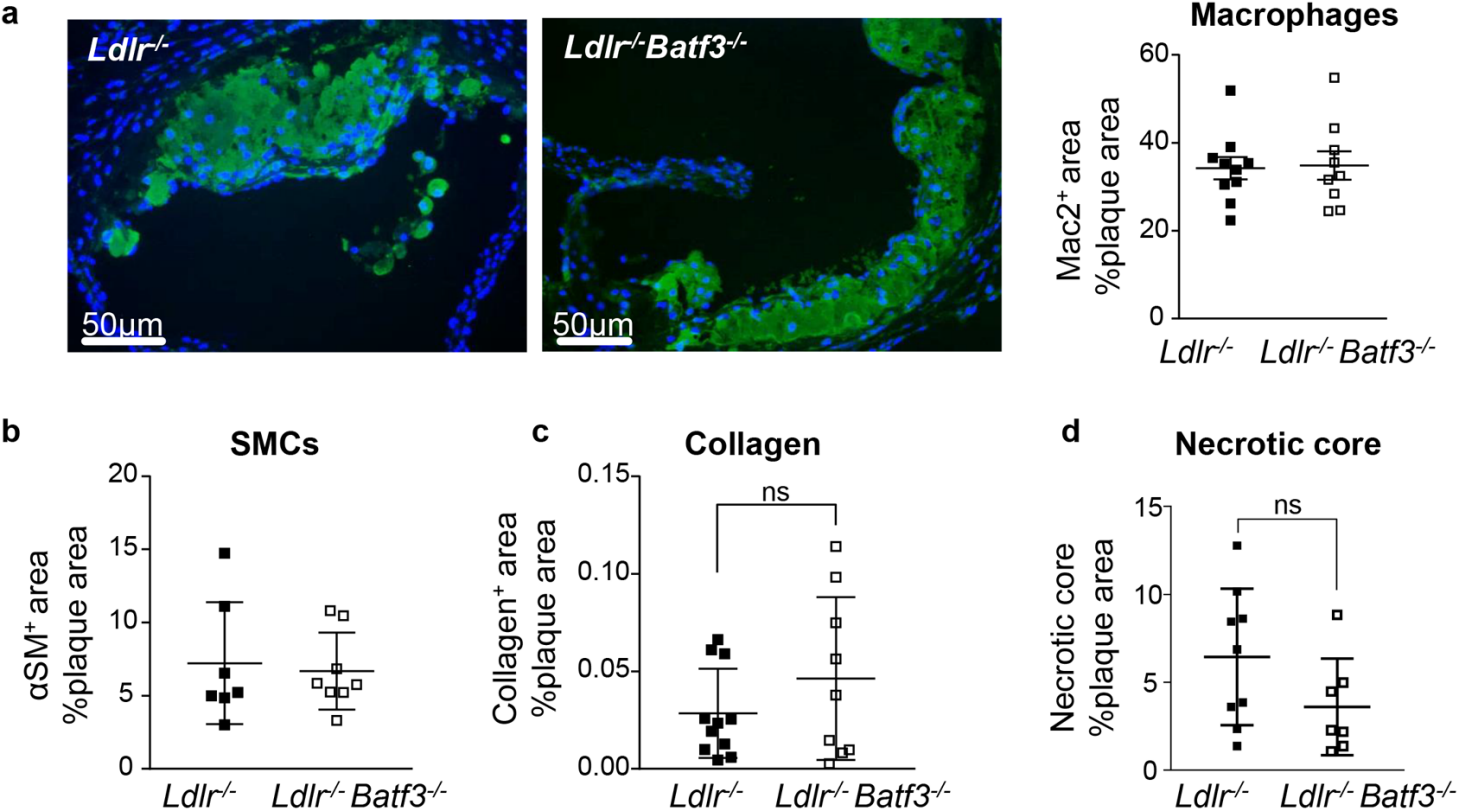
Plaque composition is not altered by *Batf3* deficiency. Quantification of the area positive for Mac-2 (a) representative images of immunofluorescence staining are shown; scale bars: 50μm; cell nuclei were counterstained with DAPI (blue), α-smooth muscle actin (b), Sirius-red (c), and of the necrotic core (d) in the aortic root from *Ldlr*^-/-^ (n = 12) and *Ldlr*^-/-^*Batf3*^-/-^ mice (n = 10) fed with 8 weeks of HFD. Data ara presented as mean ± SEM; ns, non significant.

We furthermore evaluated whether Batf3 expression affects macrophage accumulation in *Ldlr*^*-/-*^*Batf3*^*-/-*^ mice after 8 weeks of diet. Analyzing the content of F4/80^+^ CD11c^-^ macrophages by flow cytometry in the aortic sinus and aortic arch after 8 weeks of HFD, we observed no differences between groups (Panel B in S3 Fig). We further characterized the plaque phenotype after 8 weeks of HFD by immunofluorescence staining. Again, relative macrophage but also smooth muscle cell content within plaques (Fig 2A and 2B), collagen content (Fig 2C) as well as the relative necrotic core area (Fig 2D) were unaltered between groups. Similar findings were obtained when analyzing atherosclerotic lesion size and phenotype in female *Ldlr*^-/-^*Batf3*^*-/-*^ and *Ldlr*^-/-^ mice fed a HFD for 12 weeks (Panels A-E in S4 Fig). Taken together, these results indicate that Batf3 deficiency does not affect the development of atherosclerotic lesions and the plaque phenotype in both male and female *Ldlr*^-/-^ mice.

To further clarify whether Batf3 may affect macrophage foam cell formation as a key factor in atherosclerosis, we have generated bone marrow-derived macrophages (BMDMs). Compared with isolated splenic CD11c^+^ cells that contain Batf3-expressing cells, BMDMs showed only marginal *Batf3* expression (Panel C in S3 Fig). Accordingly, no differences in foam cell formation were noted in BMDMs stimulated with oxLDL for 24 hours, as revealed by flow cytometric analyzes of Nile Red staining in BMDM derived from *Ldlr*^*-/-*^ compared to *Ldlr*^*-/-*^*Batf3*^*-/-*^ mice (Panel D in S3 Fig), suggesting a minor, if any, role of Batf3 in macrophages in atherosclerosis.

### Loss of BATF3-dependent APCs mildly affects pro-atherogenic T helper cell responses

**Fig 3:**
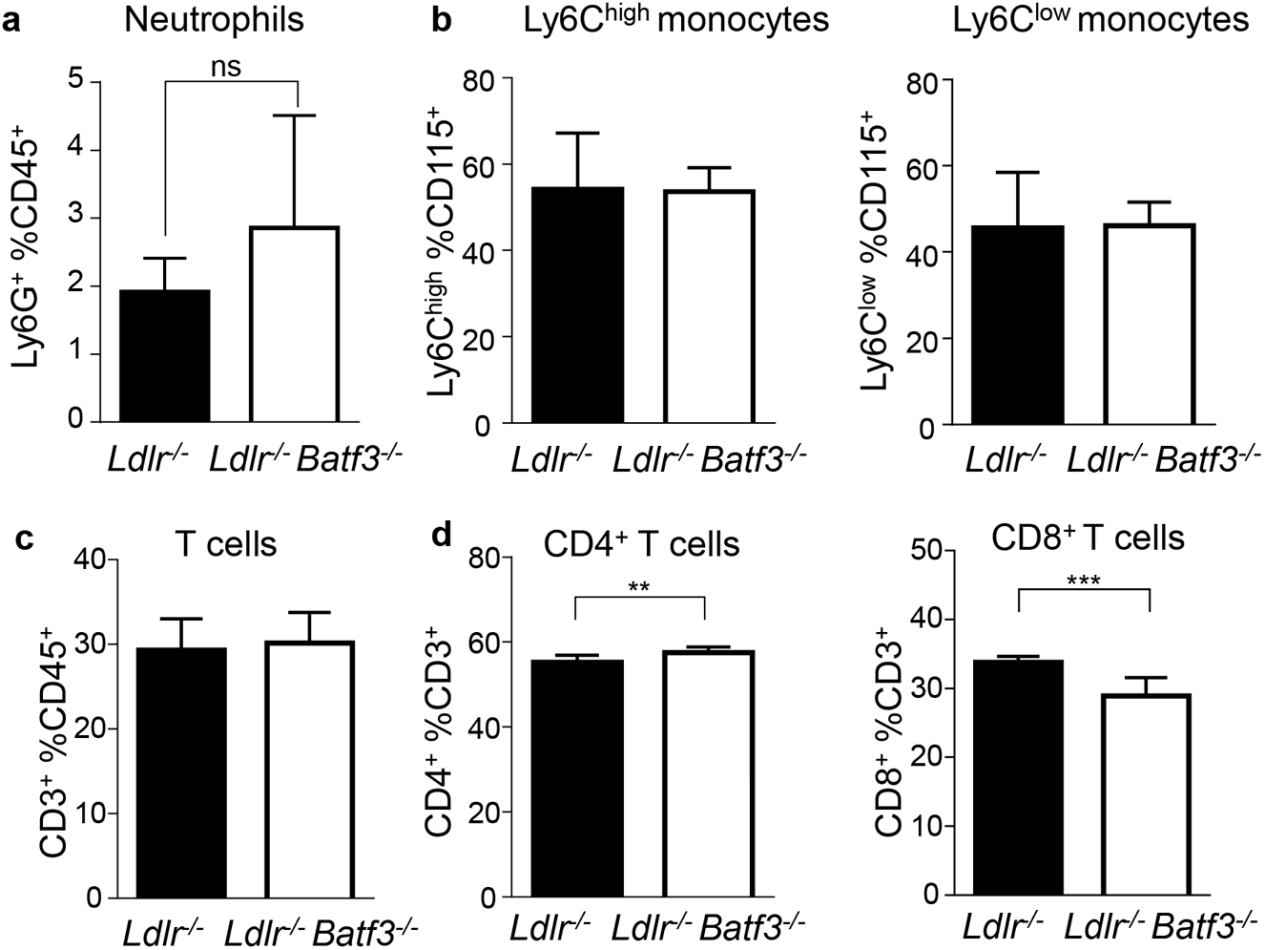
Immune responses are mildy alter in the spleen of *Batf3*-deficient mice after 8 weeks of HFD. (a-d) Flow cytometric analyses of Ly6G^+^ neutrophils (a), Ly6C^high^ and Ly6C^low^ monocytes (b), and of CD3^+^ T cells (c) among CD45^+^ leukocytes, and of frequencies of CD4^+^ and CD8^+^ T cells among CD3^+^ T cells (d) in spleens from atherosclerotic *Ldlr*^-/-^ (n = 12) and *Ldlr*^-/-^*Batf3*^-/-^ mice (n = 10) fed a HFD for 8 weeks. Data ara presented as mean ± SEM; **p<0.01; ***p<0.001; ns, non significant.

Dendritic cells play an important role in T cell priming and immune cell responses that control disease development. We therefore further assessed immune cell distributions in the blood and spleen of *Ldlr*^-/-^*Batf3*^*-/- *^and *Ldlr*^-/-^ mice fed a HFD for 8 weeks. Levels of circulating neutrophils and of Ly6C^high^ and Ly6C^low^ monocytes were similar in blood and spleen (Panels A and B in S5 Fig, and Fig 3A and 3B). Similarly, no differences in total CD3^+^ T cells and in CD4^+^ or CD8^+^ T cell distributions were detected in the blood (Panels C and D in S5 Fig), while a small but significant decrease in CD8^+^ T cell frequencies and a respective increase in CD4^+^ T cells among the total CD3^+^ T cell population was noted in the spleen (Fig 3C and 3D).

**Fig 4:**
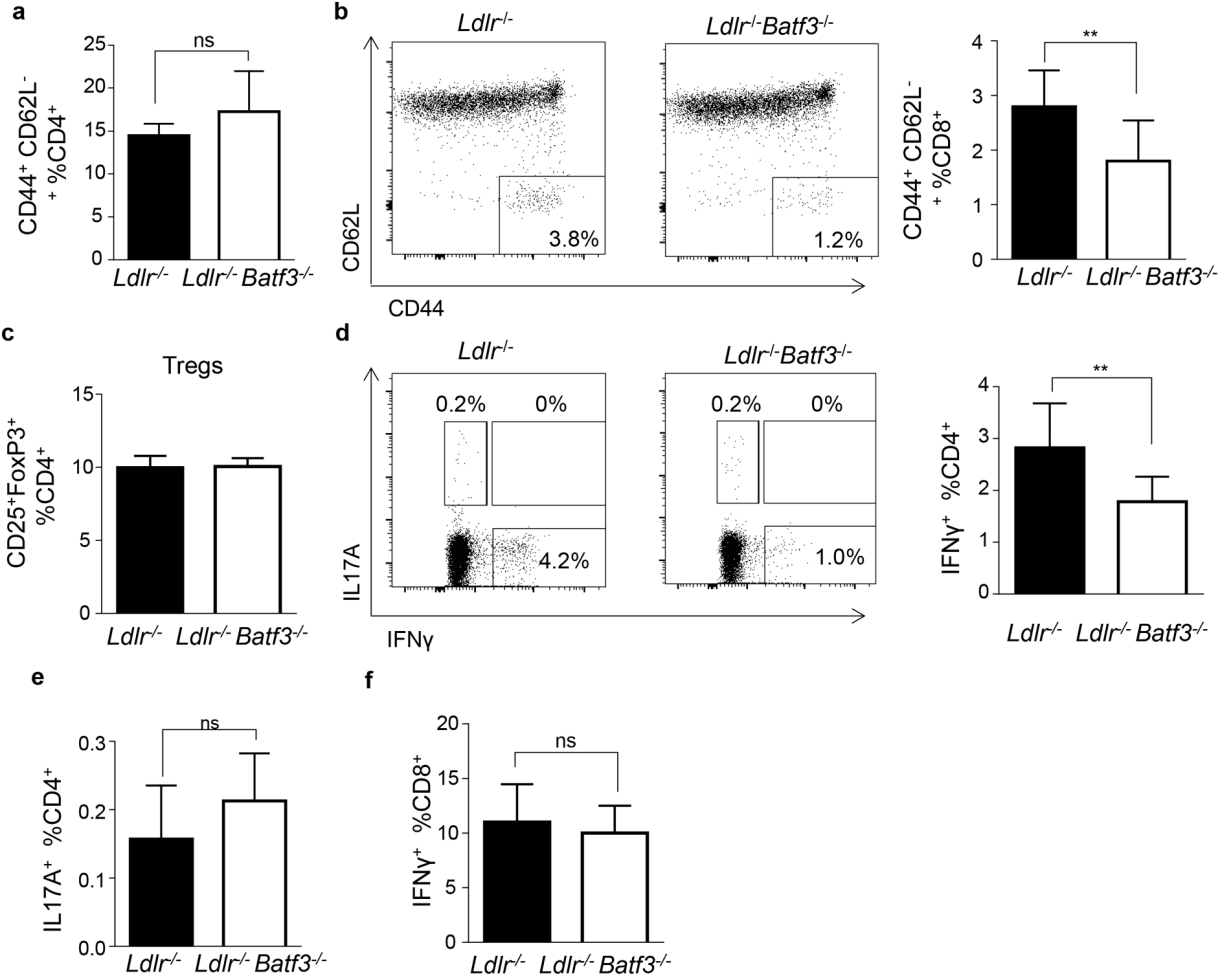
T cell activation is impaired in the spleen of *Batf3*^*-/-*^ mice after 8 weeks of HFD. Flow cytometric analyses of spleen cells obtained from atherosclerotic *Ldlr*^-/-^ (n = 12) and *Ldlr*^-/-^*Batf3*^-/-^ mice (n = 10) fed a HFD for 8 weeks. (a) Frequencies of activated CD44^high^CD62L^low^ CD4^+^ and (b) CD8^+^ T cells (representative dot plots are shown; values indicate gated events among CD4^+^ T cells). (c) Frequencies of FoxP3^+^CD25^+^CD4^+^ Tregs, (d) IFNγ^+^CD4^+^ T cells (representative dot plots are shown, values indicate gated events among CD4^+^ T cells), (e) IL-17a^+^CD4^+^ T cells and (f) IFNγ^+^CD8^+^ T cells. Data ara presented as mean ± SEM; *p<0.5;,**p<0.01; ns, non significant.

We further analyzed the activation status of T cells in the spleen. Whereas memory effector CD4^+^ T cells were unaltered, percentages of memory effector CD8^+^ T cells were reduced in *Ldlr*^-/-^*Batf3*^-/-^ compared to *Ldlr*^-/-^ *mice* (Fig 4A and 4B). Regulatory T cells (CD4^+^CD25^+^FoxP3^+^), well known to protect against atherosclerosis [24] and shown to be affected in *Flt3*^-/-^ *Ldlr*^-/-^ mice that lack CD103^+^ cells, were unaltered in the spleen of *Ldlr*^-/-^*Batf3*^-/-^ mice (Fig 4C). We furthermore noted diminished frequencies of IFNγ^+^-producing CD4^+^ T cell responses and a trend towards an increased production of IL17A^+^ CD4^+^ T cells (Fig 4E), only very few cells could be detected positive for both IFNγ and IL17A, or IL17A and IL10 (Fig 4D, and Panel A in S6 Fig). We also did not detect any differences in IL10 expression by CD4^+^ T cells (Panel A in S6 Fig).

Similar frequencies of IFNγ^+^ CD8^+^ T cells were observed between groups (Fig 4F). We have also analyzed *Tbet* and *RORgt* mRNA expression as key transcription factors defining Th1 and Th17 cells, respectively, in total spleens of *Ldlr*^*-/-*^ and *Ldlr*^*-/-*^*Batf3*^*-/-*^ mice after 8 weeks of HFD. However, we could not find any differences among groups neither in *Tbet* nor *RORgt* expression (Panel B in S6 Fig). These data suggest that Batf3-deficiency primarily affects IFNγ production by CD4^+^ T cells.

## Discussion

CD11c^+^MHCII^+^ APCs increase in numbers in atherosclerotic mice and have been considered to modulate disease development and progression by a variety of mechanisms, including polarization of T cell responses and cytokine release [5, 25] as well as modulating lipid metabolism. The use of oxLDL-pulsed antigen-presenting cells has furthermore been successfully explored as a therapeutic approach in vaccination studies and to reduce atherosclerotic lesion burden [26]. In the aorta, CD11c^+^ MHCII^+^ APCs have been reported to have a dendritic-like phenotype, with an increased ability to induce proliferation of CD4^+^ and CD8^+^ T cells and poor phagocytic capacity in comparison with CD11b^+^F4/80^+^CD11c^-^ that showed a macrophage-like phenotype [14].

In this study we aimed to clarify the exact contribution of Batf3-dependent cells in atherosclerosis that comprise both CD8α^+^ as well as CD103^+^ APC subsets. In line with previous results in *Batf3*^-/-^ mice [10], we could demonstrate an almost complete loss of CD8α^+^ APCs in the spleen of *Ldlr*^-/-^*Batf3*^-/-^ mice before and after diet. We could furthermore observe a complete loss of CD103^+^ APCs in the aortic sinus and aorta of *Ldlr*^-/-^*Batf3*^-/-^ mice fed a normal chow diet and after 8 weeks of HFD. A recent study has shown that *Batf3* is among the core genes expressed in pre-macrophages derived from yolk sac erythro-myeloid progenitors, which give raise to tissue resident macrophages that again downregulate Batf3 [27]. Although there is evidence that Batf3 is not required for macrophage differentiation [10, 21, 22], embryo-derived resident macrophages are present in the aorta [28] that could contribute to the macrophage population in atherosclerosis. An expression of Batf3 in their precursors may thus potentially also affect the abundance of resident aortic cells or their progeny in atherosclerosis lesions. However, we could not detect any differences in the content of macrophages in *Ldlr*^*-/-*^*Batf3*^*-/-*^ mice after 8 weeks of diet in the aortic sinus and aortic arch after 8 weeks of HFD. Moreover, no functional impairment in macrophage foam cell formation were observed in bone marrow-derived macrophages from *Ldlr*^*-/-*^ compared to *Ldlr*^*-/-*^*Batf3*^*-/-*^ macrophages. These results suggest that even though Batf3 might be detected in an early developmental stage of resident macrophage precursors, *Ldlr*^*-/-*^*Batf3*^*-/-*^ mice show a selective decrease in CD8α^+^ and CD103^+^ APC populations and no differences in macrophage accumulation or foam cell formation that could have contributed to atherosclerosis lesion formation. However, a cell type-restricted analyses of Batf3 deletion need to be performed to further explore a potential contribution of Batf3 expression in *bona fide* DCs and macrophages to definitively clarify a potential cell-type specific contribution.

The expansion of CD11c^+^ APCs has previously been linked to an atheroprotective decrease in plasma cholesterol levels [7]. We did not observe any differences in serum lipids between *Ldlr*^-/-^*Batf3*^-/-^ and *Ldlr*^-/-^ mice fed a high fat diet, implying that Batf3-dependent cells are not involved in cholesterol metabolism, in line with findings in chimeric *Ldlr*^-/-^ mice reconstituted with *Batf3*^-/-^ bone marrow [12], *Flt3*^-/-^ *Ldlr*^-/-^ mice that lack CD103^+^ DCs in the aorta [14] and in an *ApoE*^*-/-*^*Batf3*^*-/-*^ *mouse* model [13].

We furthermore did not observe any differences in lesion size in the aorta and aortic sinus of *Ldlr*^-/-^*Batf3*^-/-^ mice, supporting the hypothesis that CD8α/CD103^+^ APCs have no major role in atherosclerotic lesion formation. We also failed to detect any differences in the relative content of macrophages or the necrotic core area, nor in smooth muscle cell or collagen content within plaques, indicating that Batf3-dependent APCs do not modulate plaque phenotype. These results are in line with previous findings investigating atherosclerotic lesion formation and plaque composition in *Ldlr*^-/-^ mice reconstituted with *Batf3*^-/-^ bone marrow [12].

In contrast, an aggravated atherosclerotic lesion formation was observed in *Flt3*^-/-^*Ldlr*^-/-^ mice in the aortic sinus and aorta, and it was postulated that CD103^+^ in the aorta protect from atherosclerosis through a Treg-dependent mechanisms, given reduced frequencies of this protective T cell subset in vascular tissue as well as in lymph nodes and spleen [14]. Indeed, Flt3^+^ DCs seem to control Treg numbers, and CD103^+^ DCs have been shown to induce Foxp3^+^ regulatory or immune-suppressive CD4^+^ T cells also e.g. in the intestinal mucosa [29, 30]. However, Flt3 is not only expressed in this DC subset but is also present in other CD11c^+^MHCII^+^ DC subsets, and we have previously shown that deficiency in its ligand in *Flt3*^-/-^ mice for example resulted not only in a loss of CD103^+^ DCs in the aorta, but in addition was associated with a marked reduction in CD11b^+^ DCs [23]. Flt3 can also be found on some natural killer (NKs) cells [31, 32]. In our study, depletion of Batf3-dependent APC subsets did not lead to differences in Treg frequencies, further suggesting that the atheroprotective phenotype of *Flt3*^-/-^*Ldlr*^-/-^ mice may likely not have been caused by other DC subsets or cell types rather than CD103^+^ DCs.

In another study using *ApoE*^*-/-*^*Batf3*^*-/-*^ a decreased plaque size was reported after 12 weeks of HFD without alterations in Treg numbers [13]. The authors have suggested that Batf3-dependent DCs might exert their proatherogenic role via induction of Th1 responses, mainly affecting IFNγ release, which affected CCL5 secretion by macrophages, a chemokine known to promote leukocyte recruitment in plaque [33]. Although we have also observed decreased IFNγ secretion by CD4^+^ T cells we failed to detect any differences in atherosclerosis after 8 or 12 weeks of HFD. These contradictory results might have been caused by the genetic mouse model to induce hypercholesterolemia. As highlighted by Getz *et al*. [34] both *ApoE*^*-/-*^ *and Ldlr*^-/-^ mice can be useful to study the effect of a specific gene or cell population in the development of atherosclerosis as they present advantages and disadvantages due to differences in lipid distribution and effects on immune cells. In line, several studies have reported different outcomes when using *ApoE*^-/-^ or *Ldlr*^-/-^ mice [35–38].

Unaltered lesion size between groups was furthermore accompanied by unchanged numbers of circulating monocytes and neutrophils in blood and similar percentages of these immune cells in the spleen of *Ldlr*^-/-^ and *Ldlr*^-/-^*Batf3*^-/-^ mice. Similarly, total T cell numbers were unaltered. However, a decrease in the CD8^+^ T cell compartment as well as its activation status was noted in *Ldlr*^-/-^*Batf3*^-/-^ mice. This may be associated with the loss of CD8α^+^ and CD103^+^ DCs that excel in cross-presentation of antigens and activation of CD8^+^ T cells [11]. No effects on T cell activation in chimeric *Ldlr*^-/-^ mice transplanted with *Batf3*^-/-^ bone marrow [12] may have been caused by an incomplete reconstitution or additional effects on the immune system caused by irradiation, masking mild effects that we were able to uncover by using full knockout mice. Both CD103^+^ [39] and CD8α^+^ DCs [40, 41] have been reported to produce IL-12 required for Th1 responses [42] through promoting their clonal expansion, amplification and phenotypic stabilization. In line with these observations we have observed diminished IFNγ production in the spleen of *Ldlr*^-/-^*Batf3*^-/-^ mice, although we could not pinpoint Th1 T cells as the primary cell population affected, given unaltered *Tbet* expression in the spleen.

Altogether our data provide evidence that Batf3-dependent APCs do not play a major role in atherosclerotic lesion formation, but mildly affect CD8^+^ T cell activation and IFNγ production in CD4^+^ T cells. Addressing the function of single effector molecules expressed by CD8α/CD103^+^ APC^*-*^ will help to further dissect their contribution to atherosclerosis.

## Supporting information

S1 File ARRIVE guidelines checklist. (PDF)

S1 Fig *Batf3* deletion efficiently depletes CD8α^+^ and CD103^+^ APCs. Single cell suspensions from splenocytes and aortic sinus were obtained from *Ldlr*^*-*/-^ (n = 4) and *Ldlr*^*-*/-^*Batf3*^*-*/-^ (n = 4) mice fed with chow diet and analyzed by flow cytometry. (A) CD11c^+^ MHCII^+^ APCs in the spleen were gated and further discriminted by expression of CD11b and CD8α. (B) *Batf3* deletion dramatically reduced the frequency of CD8α^+^ APCs in the spleen (left) and increased CD11b^+^ APCs. (C) In the aortic sinus CD11c^+^ MHCII^+^ APCs were gated and further discriminted by expression of CD11b and CD103. CD103^+^ APCs could not be detected in the aortic sinus of *Ldlr*^*-/-*^*Batf3*^*-/-*^ mice, concomitant with an increased proportion of CD11b^+^ APCs. Data are presented as mean ± SEM; *p<0.5;***p<0.001; ns, non significant.
(PDF)

S2 Fig Lipid profiles are similar between groups after 8 weeks of HFD diet. Serum samples were fractioned to reveal VLDL, LDL and HDL content. (A) Representative lipoprotein profile and (B) serum lipid profile in *Ldlr*^*-/-*^ (n = 7) and *Ldlr*^*-/-*^*Batf3*^*-/-*^ (n = 6) mice fed a high fat diet for 8 weeks. Data are presented as mean ± SEM; ns, non significant.
(PDF)

S3 Fig *Batf3* deficiency does not affect aortic macrophage content or foam cell formation. Single cell suspensions from the aorta and aortic sinus were obtained from *Ldlr*^*-*/-^ (n = 7) and *Ldlr*^*-*/-^*Batf3*^*-*/-^ mice (n = 6) fed a HFD for 8 weeks and analyzed by flow cytometry. (A) After exclusion of CD19^+^ B cells and CD3^+^ T cells, frequencies of macrophages, defined as F4/80^+^ CD11c^-^, (B) among total CD45^+^ cells were analyzed. (C,D) BMDMs were starved with 2%BSA for 4 hours and were left either untreated or exposed to 50μg/ml of oxLDL for 24 hours. Cells were collected for qPCR analysis; *Batf3* expression in BMDMs was normalized to expression levels in purified splenic CD11c^+^ (C). Foam cell formation was analyzed using Nile Red by flow cytometry; data depict the geometric mean of Nile Red staining (D). (C) and (D) are representative experiments of a total of 3 independent experiments. Data are presented as mean ± SEM; *p<0.5; ***p<0.001; ns, non significant.
(PDF)

S4 Fig Female *Ldlr*^-/-^*Batf3*^-/-^ mice fed a HFD for 12 week showed a similar lesion size and phenotype compared to *Ldlr*^*-/-*^ mice. Total aorta and aortic sinus from female *Ldlr*^-/-^ (n = 9) and *Ldlr*^-/-^*Batf3*^-/-^ mice (n = 6) were analyzed by histology. (A) Total aorta was stained with ORO and lesion sized was determined. (B) Aortic sinus were stained with Aldehyde-fuchsine and plaque area was analyzed. Macrophage content (C), collagen (D) and smooth muscle cell content (E) was anaylzed within aortic sinus plaques by immunofluorescence. Data are presented as mean ± SEM; ns, non significant.
(PDF)

S5 Fig Neutrophil, monocyte and T cell distributions are not affected by *Batf3* deletion in atherosclerotic mice in the blood. Neutrophils, monocytes and T cells were analyzed in blood by flow cytometry. (A) CD115^-^Ly6G^+^ neutrophil, and (B) Ly6C^high^ and Ly6C^low^ CD115^+^Ly6G^-^ monocyte counts in blood. (C) Total T cells are expressed cells per μl of blood. Frequncies of CD4^+^ and CD8^+^ T cells among total CD3^+^ T cells (D). Data ara presented as mean ± SEM; ns, non significant.
(PDF)

S6 Fig Batf3-deficient mice do not show alterations in IL10 and IL17/IL10 expression in CD4^+^ T cells after 8 weeks of HFD. (A) Single cell suspensions from splenocytes were obtained from *Ldlr*^*-/-*^ (n = 7) and *Ldlr*^*-/-*^*Batf3*^*-/-*^ mice (n = 6) fed a HFD for 8 weeks and analyzed by flow cytometry. Frequencies of IL10^+^ and IL17A^+^IL10^+^ cells among CD4^+^ T cells (representative dot plots are shown, values indicate gated events among CD4^+^ T cells). (B) Total RNA was isolated from fresh-frozen spleens. Quantitative PCR results of *Tbet* and *RORγt* mRNA expression are shown. All expression levels were first normalized for levels *Hprt* expression and are depicted as fold induction when compared to expression levels in *Ldlr*^*-/-*^ animals fed a HFD for 8 weeks. Data ara presented as mean ± SEM; ns, non significant.
(PDF)
