# Supplementary material for: Deletion of Batf3-dependent antigen-presenting cells does not affect atherosclerotic lesion formation in mice
Source: PLoS One. 2017 Aug 3;12(8):e0181947. doi: 10.1371/journal.pone.0181947 (PMC5542449; doi:10.1371/journal.pone.0181947)
Supplement: S1 Fig — Single cell suspensions from splenocytes and aortic sinus were obtained from Ldlr-/- (n = 4) and Ldlr-/-Batf3-/- (n = 4) mice fed with chow diet and analyzed by flow cytometry. (A) CD11c+ MHCII+ APCs in the spleen were gated and further discriminted by expression of CD11b and CD8α. (B) Batf3 deletion dramatically reduced the frequency of CD8α+ APCs in the spleen (left) and increased CD11b+ APCs. (C) In the aortic sinus CD11c+ MHCII+ APCs were gated and further discriminted by expression of CD11b and CD103. CD103+ APCs could not be detected in the aortic sinus of Ldlr-/-Batf3-/- mice, concomitant with an increased proportion of CD11b+ APCs. Data are presented as mean ± SEM; *p<0.5;***p<0.001; ns, non significant. (PDF) [file pone.0181947.s002.pdf]

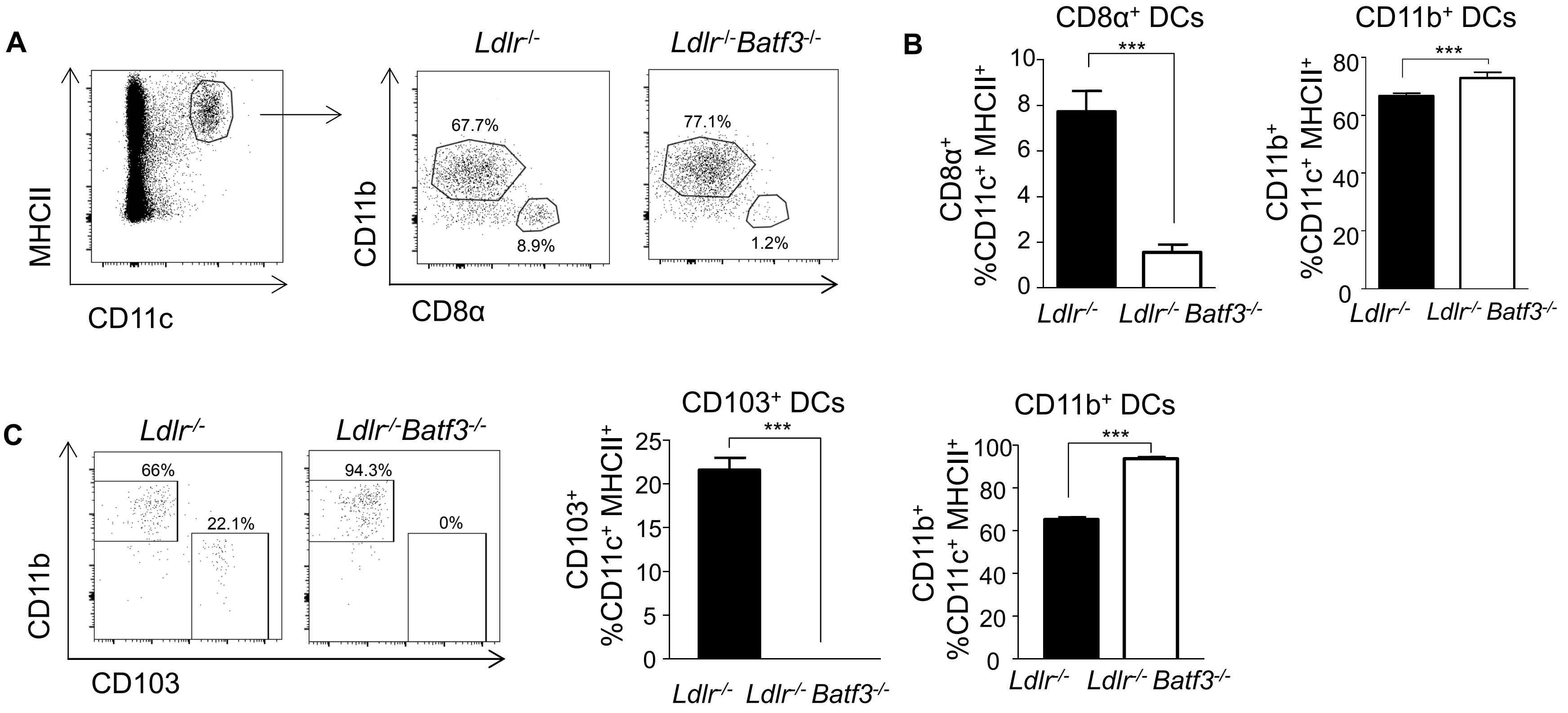

S1 Fig. *Batf3* deletion efficiently depletes CD8α$^+$ and CD103$^+$ APCs. Single cell suspensions from splenocytes and aortic sinus were obtained from *Ldlr*$^{-/-}$ (n=4) and *Ldlr*$^{-/-}$*Batf3*$^{-/-}$ (n=4) mice fed with chow diet and analyzed by flow cytometry. (A) CD11c$^+$ MHCII$^+$ APCs in the spleen were gated and further discriminted by expression of CD11b and CD8α. (B) *Batf3* deletion dramatically reduced the frequency of CD8α$^+$ APCs in the spleen (left) and increased CD11b$^+$ APCs. (C) In the aortic sinus CD11c$^+$ MHCII$^+$ APCs were gated and further discriminted by expression of CD11b and CD103. CD103$^+$ APCs could not be detected in the aortic sinus of *Ldlr*$^{-/-}$*Batf3*$^{-/-}$ mice, concomitant with an increased proportion of CD11b$^+$ APCs. Data are presented as mean ± SEM; *p<0.5;***p<0.001; ns, non significant.
