# Supplementary material for: Deletion of Batf3-dependent antigen-presenting cells does not affect atherosclerotic lesion formation in mice
Source: PLoS One. 2017 Aug 3;12(8):e0181947. doi: 10.1371/journal.pone.0181947 (PMC5542449; doi:10.1371/journal.pone.0181947)
Supplement: S2 Fig — Serum samples were fractioned to reveal VLDL, LDL and HDL content. (A) Representative lipoprotein profile and (B) serum lipid profile in Ldlr-/- (n = 7) and Ldlr-/-Batf3-/- (n = 6) mice fed a high fat diet for 8 weeks. Data are presented as mean ± SEM; ns, non significant. (PDF) [file pone.0181947.s003.pdf]

A

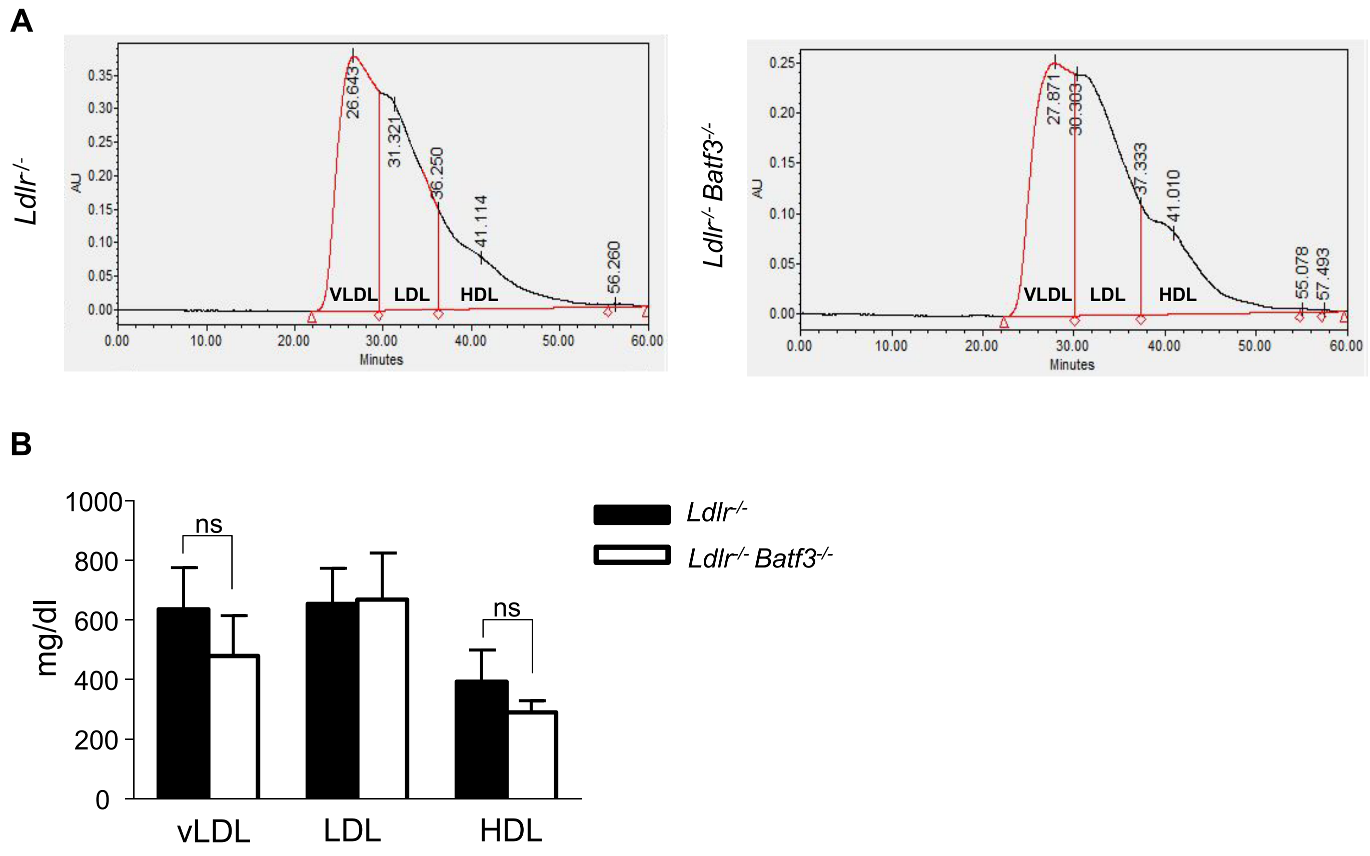

S2 Fig. Lipid profiles are similar between groups after 8 weeks of HFD diet. Serum samples were fractioned to reveal VLDL, LDL and HDL content. (A) Representative lipoprotein profile and (B) serum lipid profile in *Ldlr*$^{-/-}$ (n=7) and *Ldlr*$^{-/-}$*Batf3*$^{-/-}$ (n=6) mice fed a high fat diet for 8 weeks. Data are presented as mean ± SEM; ns, non significant.
