# Supplementary material for: Deletion of Batf3-dependent antigen-presenting cells does not affect atherosclerotic lesion formation in mice
Source: PLoS One. 2017 Aug 3;12(8):e0181947. doi: 10.1371/journal.pone.0181947 (PMC5542449; doi:10.1371/journal.pone.0181947)
Supplement: S3 Fig — Single cell suspensions from the aorta and aortic sinus were obtained from Ldlr-/- (n = 7) and Ldlr-/-Batf3-/- mice (n = 6) fed a HFD for 8 weeks and analyzed by flow cytometry. (A) After exclusion of CD19+ B cells and CD3+ T cells, frequencies of macrophages, defined as F4/80+ CD11c-, (B) among total CD45+ cells were analyzed. (C,D) BMDMs were starved with 2%BSA for 4 hours and were left either untreated or exposed to 50μg/ml of oxLDL for 24 hours. Cells were collected for qPCR analysis; Batf3 expression in BMDMs was normalized to expression levels in purified splenic CD11c+ (C). Foam cell formation was analyzed using Nile Red by flow cytometry; data depict the geometric mean of Nile Red staining (D). (C) and (D) are representative experiments of a total of 3 independent experiments. Data are presented as mean ± SEM; *p<0.5; ***p<0.001; ns, non significant. (PDF) [file pone.0181947.s004.pdf]

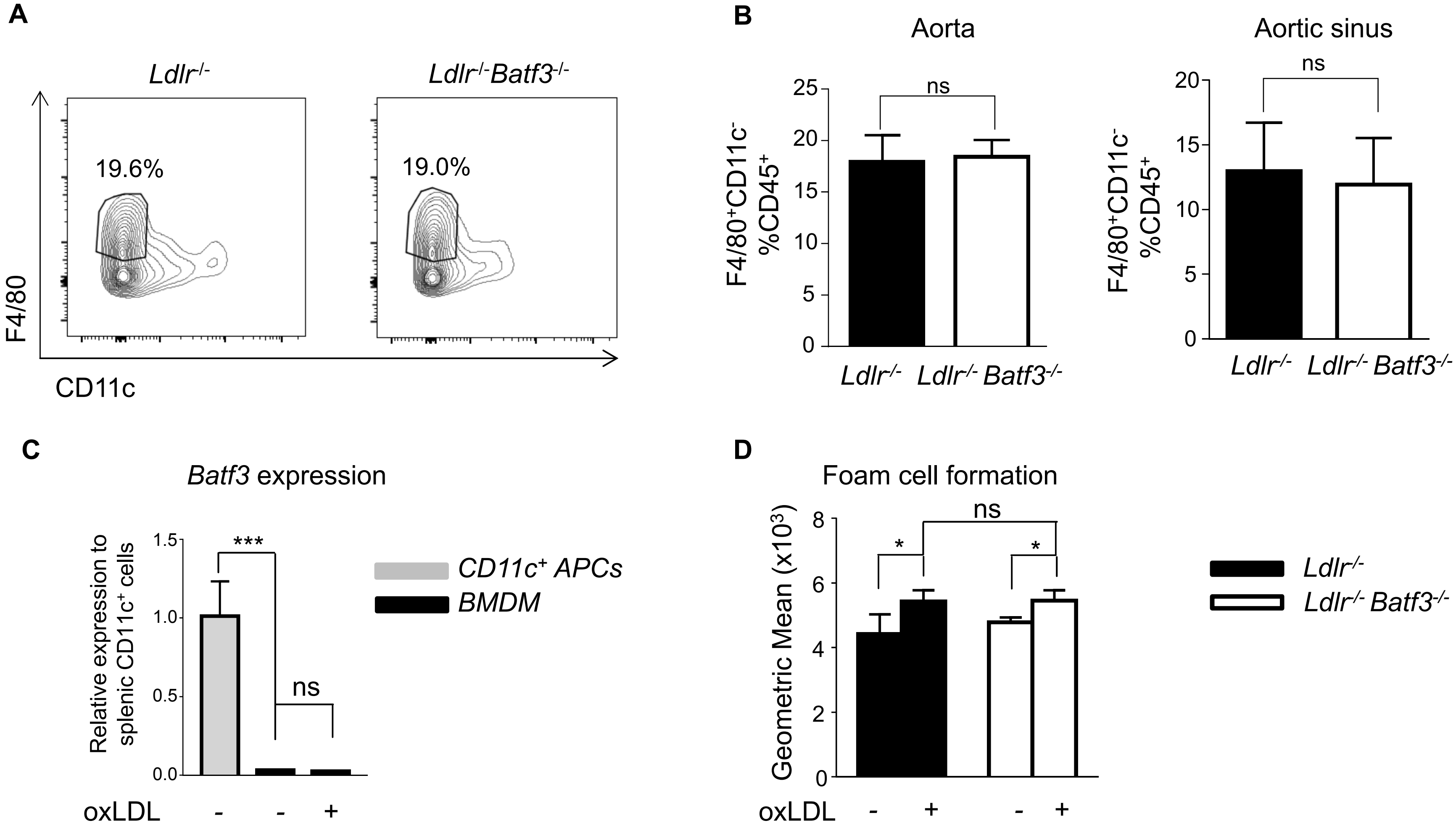

S3 Fig. *Batf3* deficiency does not affect aortic macrophage content or foam cell formation. Single cell suspensions from the aorta and aortic sinus were obtained from *Ldlr*$^{-/-}$ (n=7) and *Ldlr*$^{-/-}$*Batf3*$^{-/-}$ mice (n=6) fed a HFD for 8 weeks and analyzed by flow cytometry. (A) After exclusion of CD19^{+} B cells and CD3^{+} T cells, frequencies of macrophages, defined as F4/80^{+} CD11c^{-}, (B) among total CD45^{+} cells were analyzed. (C,D) BMDMs were starved with 2%BSA for 4 hours and were left either untreated or exposed to 50µg/ml of oxLDL for 24 hours. Cells were collected for qPCR analysis; *Batf3* expression in BMDMs was normalized to expression levels in purified splenic CD11c^{+} (C). Foam cell formation was analzyed using Nile Red by flow cytometry (D); data depict the geometric mean of Nile Red staining. (C) and (D) are representative experiments of a total of 3 independent experiments. Data are presented as mean ± SEM; *p<0.5; ***p<0.001; ns, non significant.
