# Supplementary material for: Deletion of Batf3-dependent antigen-presenting cells does not affect atherosclerotic lesion formation in mice
Source: PLoS One. 2017 Aug 3;12(8):e0181947. doi: 10.1371/journal.pone.0181947 (PMC5542449; doi:10.1371/journal.pone.0181947)
Supplement: S4 Fig — Total aorta and aortic sinus from female Ldlr-/- (n = 9) and Ldlr-/-Batf3-/- mice (n = 6) were analyzed by histology. (A) Total aorta was stained with ORO and lesion sized was determined. (B) Aortic sinus were stained with Aldehyde-fuchsine and plaque area was analyzed. Macrophage content (C), collagen (D) and smooth muscle cell content (E) was anaylzed within aortic sinus plaques by immunofluorescence. Data are presented as mean ± SEM; ns, non significant. (PDF) [file pone.0181947.s005.pdf]

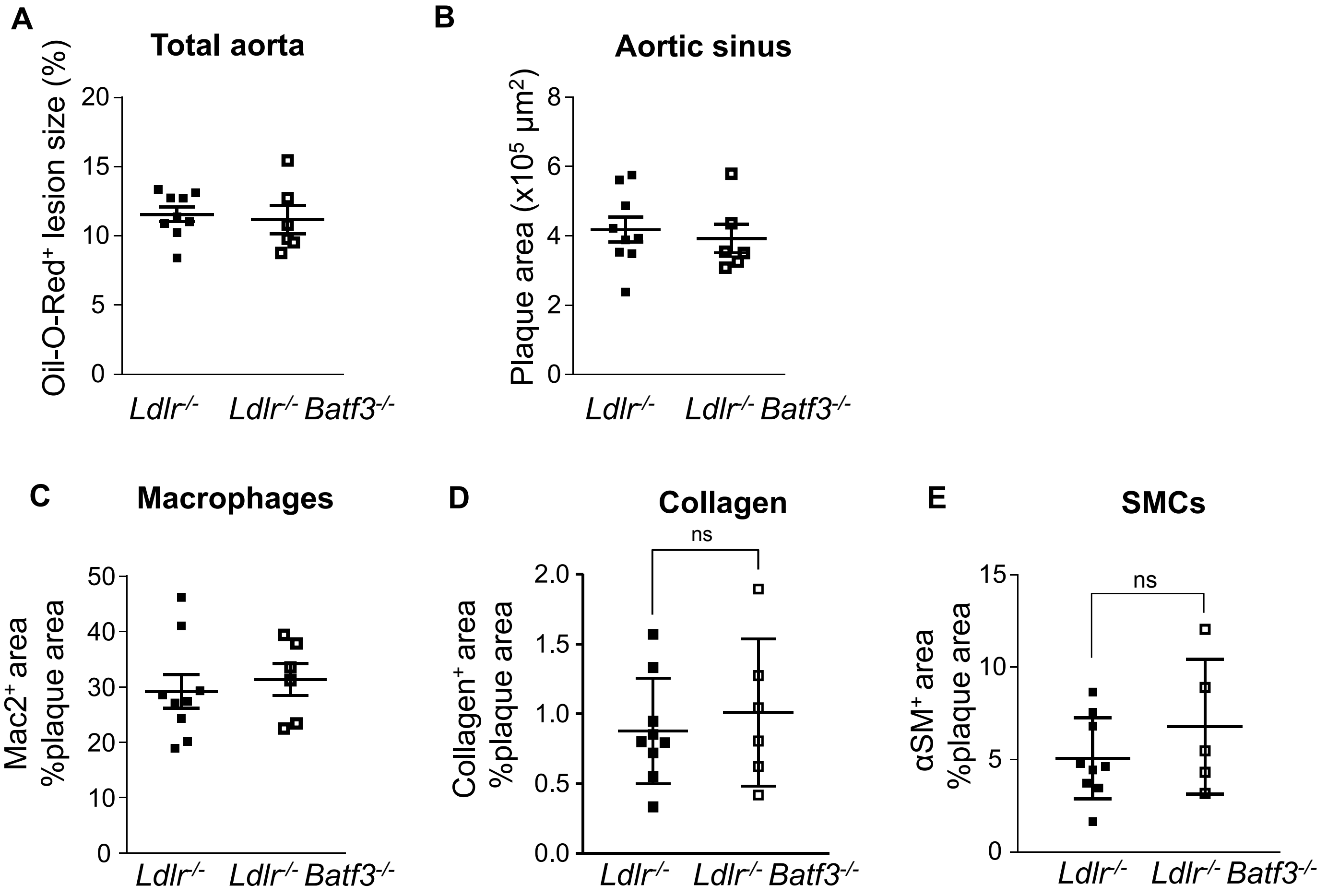

S4 Fig. Female *Ldlr$^{-/-}$Batf3$^{-/-}$* mice fed a HFD for 12 week showed a similar lesion size and phenotype compared to *Ldlr$^{-/-}$* mice. Total aorta and aortic sinus from female *Ldlr$^{-/-}$* (n = 9) and *Ldlr$^{-/-}$Batf3$^{-/-}$* mice (n = 6) were analyzed by histology. (A) Total aorta was stained with ORO and lesion sized was determined. (B) Aortic sinus were stained with Aldehyde-fuchsine and plaque area was analyzed. Macrophage content (C), collagen (D) and smooth muscle cell content (E) was anaylzed within aortic sinus plaques by immunofluorescence. Data are presented as mean ± SEM; ns, non significant.
