# Supplementary material for: Deletion of Batf3-dependent antigen-presenting cells does not affect atherosclerotic lesion formation in mice
Source: PLoS One. 2017 Aug 3;12(8):e0181947. doi: 10.1371/journal.pone.0181947 (PMC5542449; doi:10.1371/journal.pone.0181947)
Supplement: S5 Fig — Neutrophils, monocytes and T cells were analyzed in blood by flow cytometry. (A) CD115-Ly6G+ neutrophil, and (B) Ly6Chigh and Ly6Clow CD115+Ly6G- monocyte counts in blood. (C) Total T cells are expressed cells per μl of blood. Frequncies of CD4+ and CD8+ T cells among total CD3+ T cells (D). Data ara presented as mean ± SEM; ns, non significant. (PDF) [file pone.0181947.s006.pdf]

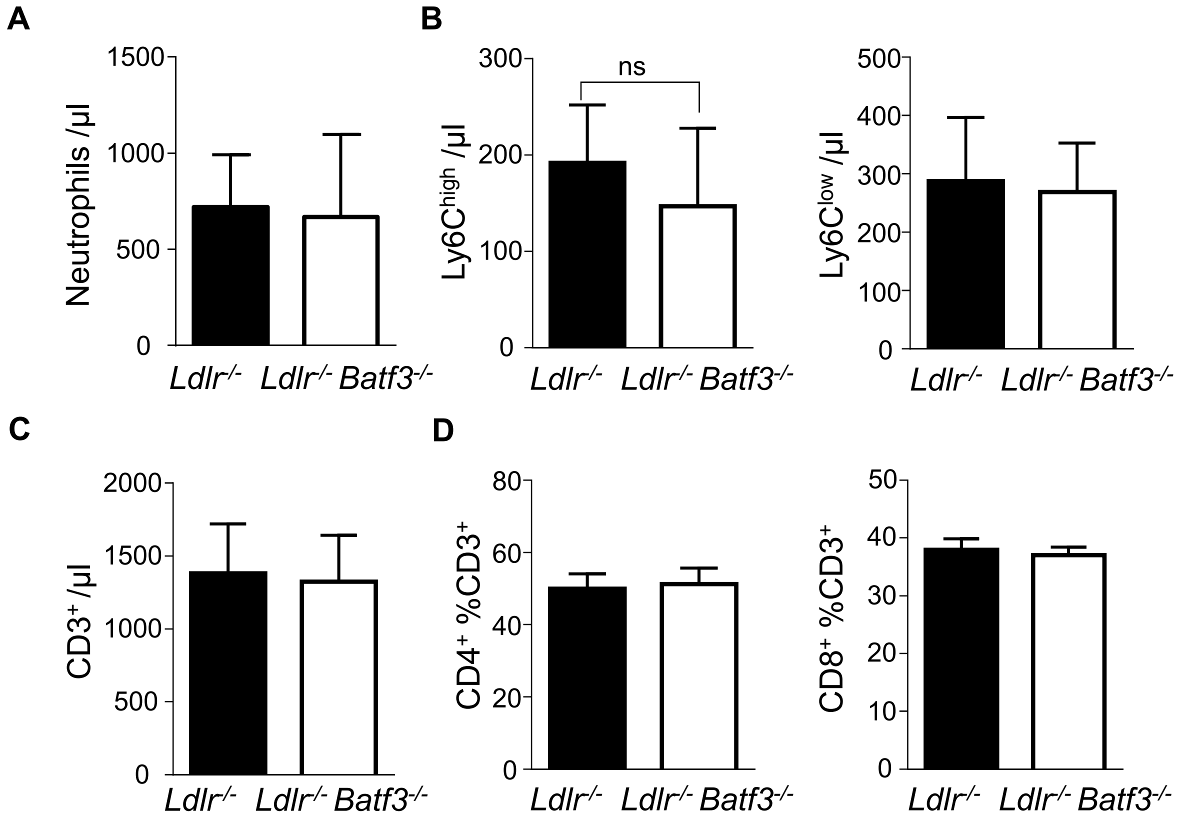

S5 Fig. Neutrophil, monocyte and T cell distributions are not affected by *Batf3* deletion in atherosclerotic mice in the blood. Neutrophils, monocytes and T cells were analyzed in blood by flow cytometry. (A) CD115$^-$Ly6G$^+$ neutrophil, and (B) Ly6C^{high} and Ly6C^{low} CD115$^+$Ly6G$^-$ monocyte counts in blood. (C) Total T cells are expressed cells per µl of blood. Frequncies of CD4$^+$ and CD8$^+$ T cells among total CD3$^+$ T cells (D). Data ara presented as mean ± SEM; ns, non significant.
