# Supplementary material for: Deletion of Batf3-dependent antigen-presenting cells does not affect atherosclerotic lesion formation in mice
Source: PLoS One. 2017 Aug 3;12(8):e0181947. doi: 10.1371/journal.pone.0181947 (PMC5542449; doi:10.1371/journal.pone.0181947)
Supplement: S6 Fig — (A) Single cell suspensions from splenocytes were obtained from Ldlr-/- (n = 7) and Ldlr-/-Batf3-/- mice (n = 6) fed a HFD for 8 weeks and analyzed by flow cytometry. Frequencies of IL10+ and IL17A+IL10+ cells among CD4+ T cells (representative dot plots are shown, values indicate gated events among CD4+ T cells). (B) Total RNA was isolated from fresh-frozen spleens. Quantitative PCR results of Tbet and RORγt mRNA expression are shown. All expression levels were first normalized for levels Hprt expression and are depicted as fold induction when compared to expression levels in Ldlr-/- animals fed a HFD for 8 weeks. Data ara presented as mean ± SEM; ns, non significant. (PDF) [file pone.0181947.s007.pdf]

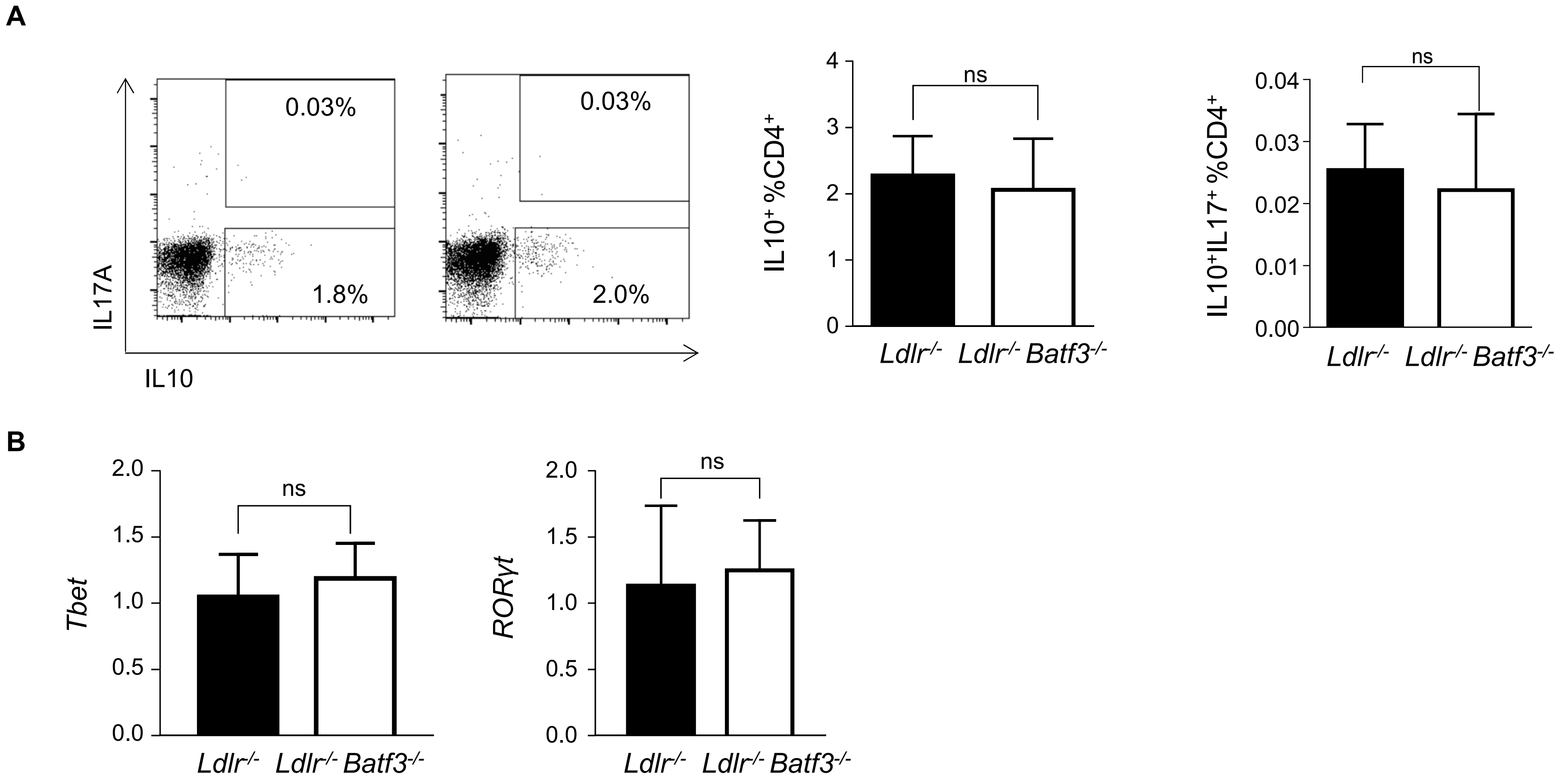

S6 Fig. Batf3-deficient mice do not show alterations in IL10 and IL17/IL10 expression in $CD4^+$ T cells after 8 weeks of HFD. (A) Single cell suspensions from splenocytes were obtained from *Ldlr*$^{-/-}$ (n=7) and *Ldlr*$^{-/-}$*Batf3*$^{-/-}$ mice (n=6) fed a HFD for 8 weeks and analyzed by flow cytometry. Frequencies of $IL10^+$ and $IL17A^+IL10^+$ cells among $CD4^+$ T cells (representative dot plots are shown, values indicate gated events among $CD4^+$ T cells). (B) Total RNA was isolated from fresh-frozen spleens. Quantitative PCR results of *Tbet* and *RORγt* mRNA expression are shown. All expression levels were first normalized for levels *Hprt* expression and are depicted as fold induction when compared to expression levels in *Ldlr*$^{-/-}$ animals fed a HFD for 8 weeks. Data ara presented as mean ± SEM; ns, non significant.
